# Cohort Profile Update: The Neuroscience in Psychiatry Network (NSPN) 2400 cohort during the COVID-19 pandemic

**DOI:** 10.1093/ije/dyad095

**Published:** 2023-07-07

**Authors:** Anna Wiedemann, Junaid Bhatti, Roxanne W Hook, Sharon A S Neufeld, NSPN Consortium, Edward Bullmore, Raymond Dolan, Ian Goodyer, Peter Fonagy, Peter Jones, Samuel Chamberlain, Michael Moutoussis, Tobias Hauser, Sharon Neufeld, Rafael Romero-Garcia, Michelle St Clair, Petra Vértes, Kirstie Whitaker, Becky Inkster, Gita Prabhu, Cinly Ooi, Umar Toseeb, Barry Widmer, Junaid Bhatti, Laura Villis, Ayesha Alrumaithi, Sarah Birt, Aislinn Bowler, Kalia Cleridou, Hina Dadabhoy, Emma Davies, Ashlyn Firkins, Sian Granville, Elizabeth Harding, Alexandra Hopkins, Daniel Isaacs, Janchai King, Danae Kokorikou, Christina Maurice, Cleo McIntosh, Jessica Memarzia, Harriet Mills, Ciara O’Donnell, Sara Pantaleone, Jenny Scott, Beatrice Kiddle, Ela Polek, , Pasco Fearon, John Suckling, Anne-Laura van Harmelen, Rogier Kievit, Richard Bethlehem, Raymond J Dolan, Peter Fonagy, Ian Goodyer, Edward T Bullmore, Samuel R Chamberlain, Peter B Jones

**Affiliations:** Department of Psychiatry, University of Cambridge, Cambridge, UK; Cambridgeshire and Peterborough NHS Foundation Trust, Cambridge, UK; National Institute for Health Research, Applied Research Collaboration, East of England, UK; Department of Psychiatry, University of Cambridge, Cambridge, UK; Department of Psychiatry, University of Cambridge, Cambridge, UK; Department of Psychiatry, University of Cambridge, Cambridge, UK; Max Planck UCL Centre for Computational Psychiatry and Ageing Research, London, UK; Research Department of Clinical, Educational and Health Psychology, University College London, London, UK; Anna Freud Centre, London, UK; Department of Psychiatry, University of Cambridge, Cambridge, UK; Department of Psychiatry, University of Cambridge, Cambridge, UK; Department of Psychiatry, Faculty of Medicine, University of Southampton, Southampton, UK; Southern Health NHS Foundation Trust, Southampton, UK; Department of Psychiatry, University of Cambridge, Cambridge, UK; Cambridgeshire and Peterborough NHS Foundation Trust, Cambridge, UK; National Institute for Health Research, Applied Research Collaboration, East of England, UK

Key Features

- The Neuroscience in Psychiatry Network (NSPN) 2400 cohort (*n* = 2403; aged 14–24 years at baseline) was conceived in 2012 to support an accelerated longitudinal design to study the emergence of psychopathology and psychiatric disorders across adolescence and young adulthood.
- Two new follow-up surveys have been established in response to the COVID-19 pandemic to assess the impact of the initial outbreak as well as any potential consequences on mental health and wellbeing within this well-defined cohort that is broadly representative of the general emerging adult population.
- The first COVID-19 follow-up was conducted between May and July 2020 and included 1000 individuals aged 19–34 years; the second COVID-19 follow-up was conducted between July and October 2022 and included 803 individuals aged 21–36 years.
- Repeated measures of psychological distress (Kessler Psychological Distress Scale; K10) and mental wellbeing (Warwick-Edinburgh Mental Wellbeing Scale; WEMWBS) were supplemented by clinical measures of depression (Patient Health Questionnaire; PHQ-9) and anxiety (Generalised Anxiety Disorder; GAD-7) to enable mapping of psychological outcomes into primary care settings such as the UK Improving Access to Psychological Therapies programme.
- The NSPN 2400 cohort has a network of existing collaborators and welcomes new collaborations, which can be directed to pbj21@cam.ac.uk. Additionally, anonymized research data are released to the global scientific research community and can be requested and downloaded through the Open: NSPN portal (https://nspn.org.uk/).

## The original cohort

The Neuroscience in Psychiatry Network (NSPN) was established in 2012 as a collaborative research initiative between the University of Cambridge and University College London. Funded by Wellcome, the cohort was conceived to support an accelerated longitudinal design to characterize normal and abnormal developmental change over the post-pubertal decade that sees the emergence of most major psychiatric disorders of adulthood.^1^ Participants were primarily recruited through general practitioners and schools in Greater London and Cambridgeshire. A total of 2403 participants were recruited into an age- and sex-stratified sample with roughly equal numbers of males and females across five age groups of 14–15, 16–17, 18–19, 20–21 and 22–24 years. The cohort was characterized in terms of psychopathological, behavioural, social and temperamental data along with DNA measurements. A subset of 785 participants completed composite cognitive tasks and clinical assessments, with 318 of these also receiving magnetic resonance imaging scans. Both subgroups provided a blood sample for genetic, epigenetic and gene expression analyses. Follow-up measurements were taken approximately 1 year later (*n* = 1836) and again 2–3 years later (*n* = 1323) if participants gave consent to be re-contacted. The last follow-up survey was completed in 2017. However, a year later all participants were invited to complete an additional survey to complete a broader range of impulsivity and compulsivity measures, some of which were not available at the time the NSPN cohort was conceived.^2^ This impulsivity and compulsivity survey was completed by 653 individuals.

## What is the reason for the new data collection?

When COVID-19 first hit the UK in February 2020 and the government subsequently announced its first national lockdown in March 2020, it was unclear how the psychological, social, educational and economic effects of stay-at-home orders would impact the mental health and wellbeing of young adults. At the time, cohort participants were still young and remained at risk for the emergence of most major psychiatric disorders of adulthood. Building on the consent to be re-contacted, we launched the first NSPN COVID-19 follow-up in May 2020. The cohort offered an ideal opportunity to study the untoward impact of the pandemic on mental health and wellbeing. All participants completed measures of psychological distress and mental wellbeing at least once prior to the COVID-19 outbreak, hence allowing us to assess potential pandemic-related changes. In addition, the cohort was designed to be broadly representative of the general population.

As the UK regained a post-pandemic equilibrium, we launched a second COVID-19 follow-up in July 2022 to assess the longer-term impact of the pandemic. In October 2022, we further invited a subset of 30 purposively sampled participants to take part in one-to-one interviews to acquire qualitative evidence of pandemic-related experiences on an individual level.

## What will be the new areas of research?

The availability of the NSPN 2400 cohort data to the global research community means that many of the original study aims are still actively researched. Both the COVID-19 follow-up surveys, however, were designed with the primary purpose of understanding the impact of the pandemic on young adults’ mental health and wellbeing. Whilst we are interested to assess the increased risk of mental ill-health within this age group, we believe it is equally important to understand who has been doing well and why. The wealth of pre-pandemic data available through NSPN will allow us to explore protective factors and potential paths of psychological resilience. In addition, recently collected interview data will provide important insights into potential mechanisms of coping and adaptation during the COVID-19 pandemic.

A further aim is to link the collected COVID-19 data to a subset of NSPN participants previously invited to study impulsivity and compulsivity traits.^2^ As maladaptive impulsive and compulsive problems can be triggered, or worsened, by stressors and isolation, we believe that assessing how the pandemic has impacted these behaviours is an important step in a relatively neglected area of mental health.

## Who is in the cohort?

**Figure 1.**
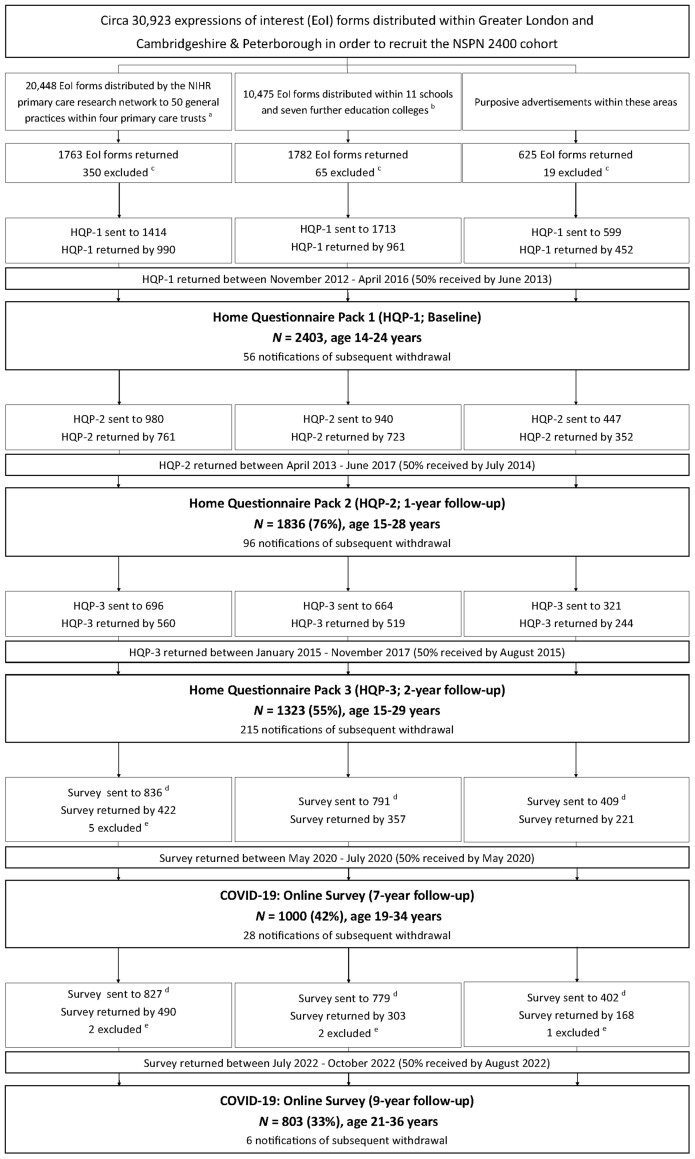
STROBE diagram. This diagram shows the recruitment stages of the Neuroscience in Psychiatry Network (NSPN) 2400 cohort. This updated diagram is based on the original cohort paper by Kiddle and colleagues (2018)^1^ and has also been published by Wiedemann and colleagues (2022).^3^ We acknowledge that there are discrepancies between the original and the updated STROBE due to data-quality checks conducted after the cohort profile was published. The updated numbers presented here or by Wiedemann and colleagues (2022)^3^ supersede the original STROBE and reflect the most accurate information available at the time of publication. EoI, Expression of Interest; HQP, Home Questionnaire Pack; ^a^36 practices in Cambridgeshire and Peterborough Primary Care Trust (PCT), 8 in Barnet PCT, 3 in Camden PCT and 3 in Islington PCT; ^b^schools in Barnet (2), Camden (4), Islington, Tower Hamlets, Haringey, Lambeth and Redbridge (all 1 each) and colleges in Cambridgeshire and Peterborough (6) and Islington (1); ^c^excluded due to current age beyond scope; ^d^both COVID-19 assessments were designed as online surveys for which all baseline participants who had a valid email address and had not withdrawn in previous assessments have been invited (note that for pre-pandemic assessments only participants who took part in the preceding round were invited); ^e^excluded due to uncertainty of survey responder identity

Based on the previously collected consent to be re-contacted for future research, ∼2000 young adults from the original NSPN 2400 cohort were invited to take part in the first and second COVID-19 surveys between May and July 2020 and July and October 2022, respectively. This represents 83% of the original cohort. We estimate that ≥5% of participants invited did not receive the invite due to outdated contact details. In the end, we received 1000 responses during the first COVID-19 follow-up in 2020 (≈53% response rate; 42% of the original cohort) and 803 responses during the second COVID-19 follow-up in 2022 (≈42% response rate; 33% of the original cohort). Participants were on average 25.6 years old (SD = 3.1 years, age range = 19–34 years) in 2020 and 27.9 years (SD = 3.1 years, age range = 21–36 years) in 2022. Please note that whilst the majority of baseline participants were recruited by June 2013, the last participant was recruited in April 2016. Therefore, some participants would have been only ∼18–19 years old during the initial COVID-19 outbreak in 2020. For further information, please consult the updated STROBE diagram in Figure 1. Overall, ∼700 individuals took part in all pre-pandemic and the first COVID-19 survey, and >400 individuals took part in all assessments, including the second COVID-19 survey, allowing modelling of individual growth curves.

We previously assessed representativeness of the cohort by comparing socio-demographic characteristics with population-based census data. This included biological sex, country of birth, ethnicity, (parental) education as well as deprivation. Table 1 provides an overview of these characteristics for both COVID-19 follow-up surveys.

**Table 1.** Socio-demographic characteristics of both Neuroscience in Psychiatry Network (NSPN) COVID-19 follow-up surveys

	Census		NSPN 2400 cohort	
Characteristic	2011	2021	COVID-19 survey 2020 (*n* = 1000)	COVID-19 survey 2022 (*n* = 803)
Age (years)				
Mean (SD)	–	–	25.6 (3.1)	27.9 (3.1)
Median (IQR)	–	–	25.0 (23.0–28.0)	28.0 (26.0–30.0)
Range	–	–	19.0–34.0	21.0–36.0
Sex (%)				
Female	50.8	51.0	63.7	64.2
Male	49.2	49.0	36.3	35.8
Country of birth (%)				
UK	86.6	83.2	86.5	86.1
Non-UK	13.4	16.8	13.4	13.8
Missing	–	–	0.1	0.1
Ethnicity (%)				
Asian or Asian British	7.5	9.3	10.3	10.1
Black, Black British, Caribbean or African	3.3	4.0	4.2	3.4
Mixed or multiple ethnic groups	2.2	2.9	6.4	6.8
White	86.0	81.7	77.8	78.7
Other ethnic group	1.0	2.1	1.3	0.7
Missing	–	–	–	0.2
Education (%)^a^				
No qualification	22.7	18.2	0.3	0.5
Vocational	3.6	5.3	2.3	2.6
Level 1–3	40.9	39.9	25.4	18.0
Level 4 or above	27.2	33.8	71.4	77.8
Other	5.7	2.8	Not assessed	Not assessed
Missing	–	–	0.6	1.0

SD, standard deviation; IQR, interquartile range; GCSE, General Certificate of Secondary Education.

a Qualification Level 1–3 includes GCSE and/or an A-level qualification at any grade. Level 4 (or above) includes at least a first degree and at most a doctoral degree. The questions regarding qualifications in the 2021 Census were significantly altered in terms of structure and content compared with the 2011 Census. These alterations partially contributed to the variations observed over the past decade. It is important to exercise caution when comparing the highest level of qualifications between 2011 and 2021, as the figures are intended solely as a reference.

Sex: Participants were originally recruited into an age- and sex-stratified sample with roughly equal numbers of males and females across five age groups of 14–15, 16–17, 18–19, 20–21 and 22–24 years. We previously reported that there was a systematic increased voluntary participation in female participants, who were over-represented by ∼5% at baseline. We observed decreased participation in males during COVID-19, resulting in a split of ∼64% female and ∼36% male participants during both most recent follow-ups.

Country of birth: Before the outbreak of the COVID-19 pandemic, the cohort closely resembled the UK population structure when looking at the proportion of UK vs non-UK births. This remained stable across all assessments with ∼86% of participants being born in the UK compared with 14% being born outside of the UK at the latest COVID-19 follow-up.

Ethnicity: The cohort broadly matched the ethnicity of the UK general population; however, we previously reported that Asian or Asian British or mixed ethnic groups were slightly over-represented. Ethnic composition across all assessments remained relatively stable with participants from a White ethnic background forming the majority with just under 80%, followed by ∼10% Asian, ∼6% mixed, ∼4% Black and ∼1% other ethnic groups.

Education: As participants of the cohort are now young adults, we no longer collected data on parental education, but on their own levels of education. We previously reported that parents of NSPN participants were more likely to complete qualifications that translated to an almost 10% difference in achieving Level 1–4 qualifications when compared with the general population. In the UK, the education system is divided into several levels, typically starting with primary education and progressing to secondary education and further education. Level 1–3 encompasses basic skills such as entry-level qualifications and the General Certificate of Secondary Education (GCSE), whilst Level 4 refers to undergraduate study at a university or higher-education institution, leading to a bachelor’s degree or equivalent qualification. The newly collected data show that participants themselves were more likely to complete qualifications. Table 1 shows that >77% achieved Level 4 (or above) qualifications at the most recent COVID-19 follow-up (participants aged between 21 and 36 years). This included at least a first degree (or equivalent) and, at most, a doctoral degree. When compared with census data, the difference is stark with only ∼27% (2011) or ∼34% (2021) of the general population obtaining Level 4 (or above) qualifications. Census 2021 qualification data by age have not yet been released, although, if we extract 2011 census data for young adults (25–34 years) only, the proportion of individuals with Level 4 (or above) qualifications increases from 27% to 40%.

**Figure 2.**
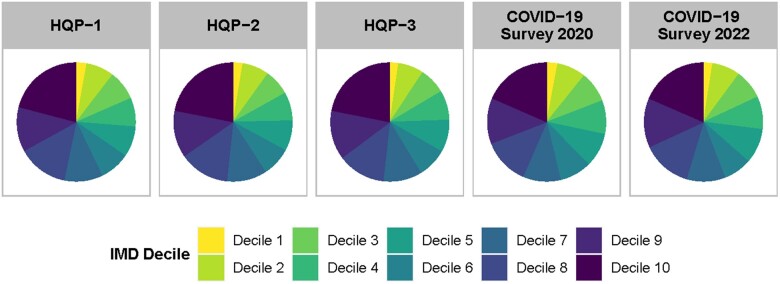
Deprivation deciles across assessments. Levels of relative deprivation amongst the Neuroscience in Psychiatry Network (NSPN) 2400 cohort participants measured by using the English Index of Multiple Deprivation (IMD; 2015). Decile 1 represents the most deprived (under-represented in NSPN) and Decile 10 represents the least deprived (over-represented in NSPN) areas across England. Note: The index combines seven domains of deprivation that include income, employment, education, health, crime, barriers to housing/services and living environment. These are used to rank each small area in England from most to least deprived with deciles being used to describe relative deprivation. HQP, Home Questionnaire Pack

Deprivation: The Index of Multiple Deprivation (IMD) was assessed via postcode at baseline. IMD has previously been calculated based on the 2010 English Indices of Deprivation, although it has since been updated to the 2015 version. This version ranks every small area in England from 1 (most deprived area) to 32 844 (least deprived area). Relative deprivation is often described in deciles whereas, for instance, the lowest decile refers to the most deprived 10% of areas in England. Figure 2 illustrates an under- and over-representation of outer deciles. For example, ∼20% of participants fall within the top decile and only ∼3% within the lowest decile compared with 10% of the general population. The NSPN 2400 cohort therefore overly represents wealthier areas of England. Nonetheless, we can observe that this remains stable over all assessments.

## What has been measured?

Table 2 lists the self-reported instruments included in the Home Questionnaire Packs (HQPs) as well as those included in the COVID-19 surveys sent in 2020 and 2022. Self-reported measures primarily focused on mood, behaviour and general wellbeing. During COVID-19, we further supplemented measures of psychological distress (Kessler Psychological Distress Scale; K10) and mental wellbeing (Warwick-Edinburgh Mental Wellbeing Scale; WEMWBS) with clinical measures of depression (Patient Health Questionnaire; PHQ-9) and anxiety (Generalised Anxiety Disorder; GAD-7) in line with current National Health Service (NHS) guidelines. Socio-demographic data collected during the pandemic included basic information such as age, gender, relationship status, highest level of educational attainment, current education or work status as well as a brief assessment of any medical conditions or other health-related information such as current or past pregnancies. All responses have then been linked to more detailed socio-demographic data from pre-pandemic assessments.

**Table 2.** List of self-reported measures available across assessment

NSPN 2400 cohort	HQP-1 baseline (*n* = 2403)	HQP-2 first follow-up (*n* = 1836)	HQP-3 second follow-up (*n* = 1323)	COVID-19 survey 2020 (*n* = 1000)	COVID-19 survey 2022 (*n* = 803)
Affective Personalities Questionnaire^6^^,^^7^^,^^a^	–	–	X	–	–
Alabama Parenting Questionnaire^8^	X	X	–	–	–
Antisocial Behaviours Checklist^a^	X	X	X	–	–
Antisocial Process Screening Device^9^	X	X	X	–	–
Barratt Impulsive Scale^10^	X	X	X	X	X
Brunnsviken Brief Quality of Life Scale^11^	–	–	–	X	–
Cambridge–Chicago Compulsivity Trait Scale^12^^,^^13^	–	–	–	X	X
Child and Adolescent Disposition Scale^14^^,^^15^	X	X	X	–	–
Community Assessment of Psychic Experiences Positive Scale^16^^,^^17^	–	–	–	X	X
Pandemic General Impact Scale^18^	–	–	–	X	–
Difficulties in Emotion Regulation Scale^19^	–	–	–	X	–
Drugs Alcohol and Self Injury^a^	X	X	X	^b^	–
Exercise Addiction Inventory^20^	–	–	–	X	X
Family Assessment Device (General Family Functioning Subscale)^21^	X	X	X	–	–
Cambridge Friendship Questionnaire^22^^,^^a^	X	X	X	–	–
Generalised Anxiety Disorder^23^	–	–	–	X	X
Impulsive-Compulsive Behaviours Checklist^24^	–	–	–	X	X
Inventory of Callous-Unemotional Traits^25^	X	X	X	–	–
Kessler Psychological Distress Scale^26^^,^^27^	X	X	X	X	X
Leyton Obsessional Inventory^28^	X	X	X	–	–
Life Events Questionnaire^29^	X	X	X	–	–
Measure of Parenting Style^30^	X	X	–	–	–
Minnesota Impulse Disorders Interview (Gambling Disorder Module)^31^	–	–	–	X	X
Moods and Feelings Questionnaire^32^	X	X	X	–	–
Padua Inventory—Washington State University Revision^33^	–	X	X	X	X
Patient Health Questionnaire^34^	–	–	–	X	X
Perceived Stress Scale^35^	–	–	–	X	–
Positive Parenting Questionnaire^a^	X	X	–	–	–
Reflective Function Questionnaire^36^	–	–	X	X	–
Revised Children’s Manifest Anxiety Scale^37^	X	X	X	–	–
Rosenberg Self-Esteem Scale^38^	X	X	X	–	–
Schizotypal Personality Questionnaire^39^	X	X	X	–	–
SCOFF Questionnaire^40^	–	–	–	X	X
Short UPPS-P Impulsive Behaviour Scale^41^	–	–	–	X	–
Technology Questionnaire^18^	–	–	–	X	–
UCLA Three Item Loneliness Scale^42^	–	–	–	–	X
Warwick-Edinburgh Mental Well-being Scale^43^	X	X	X	X	X
WHO Adult ADHD Self-Report Scale^44^	–	–	–	X	–
Young’s Internet Addiction Test^45^	–	–	–	X	X

NSPN, Neuroscience in Psychiatry Network; HQP, Home Questionnaire Pack; SCOFF, Sick, Control, One Stone, Fat, Food; UPPS-P, Urgency, Premeditation, Perseverance, Sensation Seeking, and Positive Urgency; UCLA, University of California, Los Angeles; WHO, World Health Organization; ADHD, Attention-Deficit/Hyperactivity Disorder.

a The questionnaire was designed for the sole purpose of the study; if accompanied by a reference, questions were slightly altered from those in the original measure.

b The original questionnaire has been replaced by separate questionnaires, as well as questions on illicit drugs and sexual behaviour and an abridged version of previously asked self-harm questions.

Both recent surveys included a range of pandemic-related questions designed for the sole purpose of this study including, but not limited to, information about the current living situation, childcare commitments, pandemic-related adverse experiences, workability, putative COVID-19 infection and symptoms, and, during the latest follow-up, details on COVID-19 vaccination status and confirmed infections and recovery. A list of pandemic-related questions can be found in the Supplementary material (available as Supplementary data at *IJE* online).

## What has it found? Key findings and publications

Over 50 papers have been published since the inception of the NSPN 2400 cohort (https://nspn.org.uk/publications/) focusing on the different domains of data collection including cognitive, structural and functional neuroimaging, phenomenology and epidemiology. Analyses of the COVID-19 data are largely ongoing; however, we provide a brief summary of the initial findings below.

During the initial outbreak of the COVID-19 pandemic in the UK, we saw a significant decline in mental wellbeing and an increase in psychological distress.^3^ Approximately 30% of young adults experienced symptoms of clinical depression or anxiety according to NHS guidelines and 20% had symptoms of both. The pandemic and lockdown affected young adults across the board, regardless of pre-existing risk factors. However, young adults with pre-existing mental health conditions (predominantly an existing diagnosis of depression or anxiety) were more vulnerable to increased psychological distress, even when taking their pre-pandemic mental health into account. These findings highlight the importance of maintaining access to mental healthcare services during future pandemics or lockdowns.

We further found that factors that were previously thought to enhance resilience at the individual, family and community levels (assessed through self-report at the beginning of the study) did not protect against the psychological impacts of the pandemic, although some factors did show small effects.^3^ This indicates that socio-environmental factors that typically support mental health, particularly in response to adverse events, were only mildly effective in helping individuals to cope with the mental health effects of lockdown or other aspects of the pandemic.

We also identified prior symptoms of disordered eating as the strongest predictor of suffering from an eating disorder during the initial outbreak of the COVID-19 pandemic, whilst also highlighting the significance of a history of low sensation seeking impulsivity, concurrent lack of perseverance and conflict at home as further important predictors.^4^ This highlights the importance of impulsive traits and the immediate environment such as family dynamics as critical contributors to eating disorder symptomatology in the context of the pandemic.

**Figure 3.**
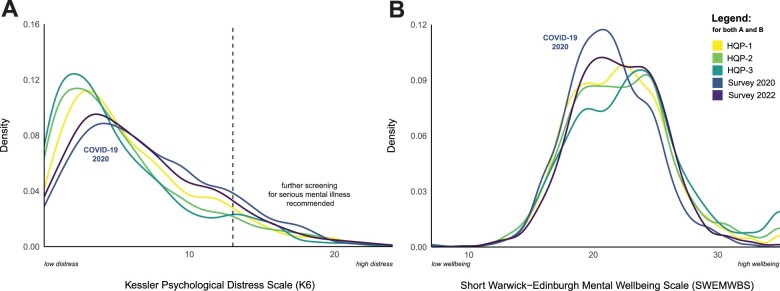
Psychological distress and mental wellbeing across assessments. Comparison of density distributions for the Kessler Psychological Distress Scale (A) and the Short Warwick-Edinburgh Mental Wellbeing Scale (B) across all Neuroscience in Psychiatry (NSPN) 2400 cohort assessments including both COVID-19 follow-up surveys in 2020 and 2022. HQP, Home Questionnaire Pack

More recently collected data show that levels of psychological distress remained elevated 2.5 years after the initial COVID-19 outbreak. Levels of mental wellbeing, however, returned to pre-pandemic levels, suggesting that psychological distress and mental wellbeing may measure distinct constructs and should not be considered uncritically as being at different ends of a single mental health continuum. Figure 3 shows the corresponding density distributions for all five NSPN assessments from 2012 to 2022. It can also be seen that levels of psychological distress continuously decreased and mental wellbeing continuously increased before the COVID-19 pandemic. This is in line with previous longitudinal research, reflecting an improvement of general psychological wellbeing when transitioning from adolescence to adulthood.^5^

## What are the main strengths and weaknesses?

One of the biggest strengths of this cohort is the availability of high-quality mental health data before the outbreak of the COVID-19 pandemic. This is further consolidated through the use of various standardized measures that are known to be reliable and stable over time. The wealth of pre-pandemic data ranging from behavioural, cognitive and neuroimaging data provide ample opportunity to be combined with mental health outcomes during the pandemic. Due to the nature of the accelerated longitudinal design of the study, pre-pandemic and pandemic developmental trajectories overlap; pre-pandemic ones can be obtained from the ages of 14–29 years and pandemic-related ones from the ages of 19–36 years.

The current sample is affected by attrition as expected in longitudinal studies. Unfortunately, we lost significantly more male compared with female participants during the most recent surveys, potentially limiting the generalizability to the larger young adult population. Furthermore, the cohort is more highly educated when compared with the general population and those from the most deprived areas are under-represented—although the latter were already the case at baseline. Fortunately, ethnic groups as well as the proportion of participants born in and outside of the UK remain representative and stable across the latest follow-up surveys. Furthermore, we acknowledge that the gap between data collected during the pandemic and data obtained prior to the pandemic is not optimal but using historical data within the COVID-19 context seems reasonable, although availability closer to the pandemic would have been ideal. Despite these shortcomings we believe that this cohort offers an important data source when studying the impact of the COVID-19 pandemic and offers the availability of high-quality data to the global research community.

## Can I get hold of the data? Where can I find out more?

The study is committed to open science. Participants have consented to their de-identified data being made available to other researchers. To date, pre-pandemic anonymized data are fully available to the research community and can be requested and downloaded through the Open: NSPN portal by following this link: https://nspn.org.uk/. Both COVID-19 data sets are planned to be made available in the same way and can be requested from the senior author Prof Peter B. Jones (pbj21@cam.ac.uk) in the meantime.

## NSPN consortium

Chief/Principal Investigators: Edward Bullmore^1,4,5^, Raymond Dolan^2,6^, Ian Goodyer^1^, Peter Fonagy^3^, Peter Jones^1^, Samuel Chamberlain^1^; NSPN (funded) Staff: Michael Moutoussis^2,6^, Tobias Hauser^2,6^, Sharon Neufeld^1^, Rafael Romero-Garcia^1,4^, Michelle St Clair^1^, Petra Vértes^1,4^, Kirstie Whitaker^1,4^, Becky Inkster^1^, Gita Prabhu^2,6^, Cinly Ooi^1^, Umar Toseeb^1^, Barry Widmer^1^, Junaid Bhatti^1^, Laura Villis^1^, Ayesha Alrumaithi^1^, Sarah Birt^1^, Aislinn Bowler^6^, Kalia Cleridou^6^, Hina Dadabhoy^6^, Emma Davies^1^, Ashlyn Firkins^1^, Sian Granville^6^, Elizabeth Harding^6^, Alexandra Hopkins^2,6^, Daniel Isaacs^6^, Janchai King^6^, Danae Kokorikou^3,6^, Christina Maurice^1^, Cleo McIntosh^1^, Jessica Memarzia^1^, Harriet Mills^6^, Ciara O’Donnell^1^, Sara Pantaleone^6^, Jenny Scott^1^, Beatrice Kiddle^1^, Ela Polek^1^; Affiliated Scientists: Pasco Fearon^3^, John Suckling^1^, Anne-Laura van Harmelen^1^, Rogier Kievit^2,7^, Richard Bethlehem^1^.

^1^Department of Psychiatry, University of Cambridge, Cambridge, UK,

^2^Max Planck UCL Centre for Computational Psychiatry and Ageing Research, London, UK,

^3^Research Department of Clinical, Educational and Health Psychology, University College London, London, UK,

^4^Behavioural and Clinical Neuroscience Institute, University of Cambridge, Cambridge, UK,

^5^ImmunoPsychiatry, GlaxoSmithKline Research and Development, Stevenage, UK,

^6^Wellcome Centre for Human Neuroimaging, University College London, London, UK and

^7^Medical Research Council Cognition and Brain Sciences Unit, University of Cambridge, Cambridge, UK

## Ethics approval

Ethical approval was granted by the Cambridge East Research Ethics Committee under REC 12/EE/0250 for all pre-pandemic assessments and REC 16/EE/0260 for both COVID-19 assessments.

## Supplementary Material

dyad095_Supplementary_Data

## Data Availability

See ‘Can I get hold of the data?’ above.
