# Correction to: Cohort Profile Update: The Neuroscience in Psychiatry Network (NSPN) 2400 cohort during the COVID-19 pandemic

**DOI:** 10.1093/ije/dyae157

**Published:** 2024-11-12

This is a correction to: Anna Wiedemann, Junaid Bhatti, Roxanne W Hook, Sharon A S Neufeld, NSPN Consortium, Raymond J Dolan, Peter Fonagy, Ian Goodyer, Edward T Bullmore, Samuel R Chamberlain, Peter B Jones, Cohort Profile Update: The Neuroscience in Psychiatry Network (NSPN) 2400 cohort during the COVID-19 pandemic, *International Journal of Epidemiology*, Volume 52, Issue 6, December 2023, Pages e315–e323, https://doi.org/10.1093/ije/dyad095

The Funding section of this paper has been corrected to include the following statement:

“The NSPN COVID-19 2020 follow-up survey included a collection of items derived from questionnaire material developed as part of the COVID-19 Social Study, which was supported by the Nuffield Foundation (WEL/FR-000022583), the MARCH Mental Health Network funded by the Cross-Disciplinary Mental Health Network Plus initiative supported by UK Research and Innovation (ES/S002588/1), and the Wellcome Trust (221400/Z/20/Z and 205407/Z/16/Z).”

