# Supplementary material for: Cohort Profile Update: The Neuroscience in Psychiatry Network (NSPN) 2400 cohort during the COVID-19 pandemic
Source: Int J Epidemiol. 2023 Jul 7;52(6):e315–23. doi: 10.1093/ije/dyad095 (PMC10749753; doi:10.1093/ije/dyad095)
Supplement: dyad095_Supplementary_Data [file dyad095_supplementary_data.pdf]

Supplementary Materials

Cohort Profile Update: The Neuroscience in Psychiatry Network (NSPN) 2400 Cohort during the COVID-19 pandemic

Anna Wiedemann[1,2,3], Junaid Bhatti[1], Roxanne W Hook[1], Sharon AS Neufeld[1], NSPN Consortium*, Raymond J Dolan[4], Peter Fonagy[5,6], Ian Goodyer[1], Edward T Bullmore[1], Samuel R Chamberlain[7,8], and Peter B Jones[1,2,3]

[1] Department of Psychiatry, University of Cambridge, UK
[2] Cambridgeshire and Peterborough NHS Foundation Trust, UK
[3] National Institute for Health Research, Applied Research Collaboration, East of England, UK
[4] Max Planck UCL Centre for Computational Psychiatry and Ageing Research, UK
[5] Research Department of Clinical, Educational and Health Psychology, University College London, UK
[6] Anna Freud Centre, London, UK
[7] Department of Psychiatry, Faculty of Medicine, University of Southampton, UK
[8] Southern Health NHS Foundation Trust, UK

*A list of authors and their affiliations can be found at the end of the main article.

Corresponding author: Anna Wiedemann, Department of Psychiatry, University of Cambridge, Douglas House, 18B Trumpington Road, Cambridge, CB2 8AH, United Kingdom,
Email: aw778@medschl.cam.ac.uk

NSPN COVID-19 Questionnaire 2020

Living Situation

During the COVID-19 lockdown which option best describes your main living situation?

- Living alone
- Living with partner
- Living with partner and other family member(s)
- Living with other family member(s)
- Living with friend(s)
- Living in shared accommodation
- Other

Childcare Commitments

During the COVID-19 lockdown have you had major childcare commitments (e.g., looking after your child/children, or other people's child/children, in your home)? [Yes/No]

Pandemic Adverse Experience

Have you experienced any of the following in the past month? Tick all that apply.

- Lost your job/been unable to do paid work
- Your spouse/partner lost their job or was unable to do paid work
- Major cut in household income (e.g., due to you or your partner being furloughed/put on leave/not receiving sufficient work)
- Unable to pay bills/rent/mortgage
- Evicted/lost accommodation
- Unable to access sufficient food
- Unable to access required medication
- Somebody close to you is ill in hospital (due to COVID-19 or another illness)
- You lost somebody close to you (due to COVID-19 or another cause)
- None of the above

Workability

Assume that your work ability at its best has a value of 10 points and 10 means you can work at the best you would ever be able to. How many points would you give your current work ability (0 means that you cannot currently work at all)? For this question, please assume that there is an opportunity to work that is suitable for your skills and background. [Scale 1-10]

Has your income been affected by the global pandemic? [Yes/No]

If yes, please specify which:

- Loss of job
- Reduction of income
- Being furloughed
- Other (e.g., reduced physical or mental capacity to work)

Infection

Since the COVID-19 outbreak, I have felt unwell and wonder if it could be (or could have been) the virus. [Yes/No]

[If relevant] At the time when you felt most unwell, and thought it could be COVID-19, tick any symptoms you experienced.

- Temperature
- Excessive Sweating (e.g., finding bedsheets soaked with sweat at night)
- Not being able to smell things
- Dry Cough
- Wet Cough (i.e., coughing up sputum, also known as productive cough)
- Headache
- Diarrhoea
- Being physically sick (vomiting)
- Short of breath when doing activities
- Short of breath when resting (such as lying down or sitting down)
- Muscle ache
- Fatigue
- Runny nose
- Loss of appetite

Have you had a test for COVID-19? [Yes/No]

Was it positive or negative?

- Positive
- Negative

Has a doctor or nurse told you you've got COVID-19? [Yes/No]

Isolation

What is your current isolation status? Select the option that best describes you.

- I am in full isolation, not leaving my home at all.
- I am staying at home, only leaving for exercise, food shopping or accessing medication, or limited social contact allowed by government (e.g., meeting one other person but staying 2 meters away).
- I am staying at home, only leaving for exercise, food shopping or accessing medication, or limited social contact allowed by government (e.g., meeting one other person but staying 2 meters away) <u>and</u> work (e.g., as a key worker or volunteer).
- I am <u>not</u> following the stay-at-home and social contact recommendations but am adhering to social distancing when in public (e.g., staying 2 meters away from others).
- I am <u>not</u> following the stay-at-home recommendations or social distancing when I am out.

Public Health Compliance

Are you following recommendations from authorities to prevent the spread of COVID-19? [7-point Likert Scale]

Other

In the past 7 days, how many days have you: [Scale 1-7]

1. Been self-isolating (not leaving the house)?
2. Been outside for 15 minutes or more?
3. Had face-to-face contact with another person for 15 minutes or more (including someone you live with)?
4. Had a phone or video call with another person for 15 minutes or more?

In the past 7 days have you done any of the following to support your mental health? Tick any that apply.

- Taken medication (e.g., anti-depressants)
- Spoken with a psychiatrist, psychologist, or other mental health professional
- Spoken with a GP or other healthcare professional about your mental health
- Spoken to somebody on a support helpline (e.g., Samaritans or NHS Volunteers)
- Accessed an online mental health programme (e.g., CBT)
- Spoken with others on an online mental health forum
- Used other mental health resources (e.g., self-help books, videos, or apps)
- Spent time on self-care specifically to help your mental health (e.g., mindfulness, meditation, or planning time for hobbies or relaxation)
- Spoken about your mental health to a friend or family member
- None of the above

NSPN COVID-19 Questionnaire 2022

Living Situation

Which option best describes your main living situation?

- Living alone
- Living with partner
- Living with partner and other family member(s)
- Living with other family member(s)
- Living with friend(s)
- Living in shared accommodation
- Other

Were there any changes in your living situation over the last two years? [Yes/No]

If yes, how would you describe your primary living situation over the last two years?

- Living alone
- Living with partner
- Living with partner and other family member(s)
- Living with other family member(s)
- Living with friend(s)
- Living in shared accommodation
- Other

Childcare Commitments

Do you currently have any major childcare commitments? (e.g., looking after your child/children, or other people's child/children, in your home) [Yes/No]

Were there any significant changes in your childcare commitments over the last two years? Please tick 'yes' if you don't have any childcare commitments now but had significant childcare commitments in the last two years. [Yes/No]

If yes, compared to your current childcare commitments, did you have more or less childcare commitments over the last two years?

- Less
- More

Pandemic Adverse Experience

Over the last two years, have you experienced any of the following? Tick all that apply.

- Lost your job/been unable to do paid work
- Your spouse/partner lost their job or was unable to do paid work
- Major cut in household income (e.g., due to you or your partner being furloughed/put on leave/not receiving sufficient work)
- Unable to pay bills/rent/mortgage
- Evicted/lost accommodation

- Unable to access sufficient food
- Unable to access required medication
- Somebody close to you is ill in hospital (due to COVID-19 or another illness)
- You lost somebody close to you (due to COVID-19 or another cause)
- None of the above

Workability

Assume that your work ability at its best has a value of 10 points and 10 means you can work at the best you would ever be able to. How many points would you give your current work ability (0 means that you cannot currently work at all)? For this question, please assume that there is an opportunity to work that is suitable for your skills and background. [Scale 1-10]

Vaccination

Which option best describes your COVID-19 vaccine status? Please note that *fully vaccinated* means you have received two doses of an approved vaccine (e.g., Oxford/AstraZeneca, Pfizer/BioNTech, Moderna etc.), or a single-dose Janssen/Johnson & Johnson vaccine.

- I am fully vaccinated and have received a booster.
- I am fully vaccinated but have not received a booster.
- I am partially vaccinated, e.g., I have only received one dose of the Oxford/AstraZeneca, Pfizer/BioNTech, Moderna etc. vaccine.
- I am exempt from vaccination on the basis of a medical exemption.
- I am not currently vaccinated.

[If relevant] Please evaluate the severity of side effects that occurred after receiving your COVID-19 vaccine where 1 means no (or negligible) side effects and 10 means very high severity. [Scale 1-10]

- First Vaccination
- Second Vaccination
- Booster

[If relevant] Please evaluate the level of fear accompanying the side effects that occurred after receiving your COVID-19 vaccine where where 1 means no (or very low) level of fear and 10 means very high level of fear. [Scale 1-10]

- First Vaccination
- Second Vaccination
- Booster

Infection

Have you been infected with the COVID-19 virus?

- No, not that I am aware
- Yes, confirmed by a positive test
- Yes, based on medical advice
- Yes, based on strong personal suspicion
- Unsure

[If relevant] Have you been infected with the COVID-19 virus more than once?

- Yes
- No
- Unsure

[If relevant] When, or when do you think, were you infected. Tick all that apply.

- ▪ Prior to vaccination; or with no vaccination if unvaccinated
- ▪ Between my 1st and 2nd dose of vaccine (for those vaccinated with BioNTech/Pfizer, Moderna or AstraZeneca vaccines)
- ▪ After receiving all required doses (two doses of BioNTech/Pfizer, Moderna or AstraZeneca vaccines or a single dose of Janssen/Johnson & Johnson vaccine)

[If relevant] How long have you had/did you have COVID-19 symptoms overall. Please include time spent with mild symptoms and the time in between symptoms if these have been coming and going. If you have caught COVID-19 more than once, please answer about the longest episode of illness you experienced.

- Less than 2 weeks
- 2 - 3 weeks
- 4 - 12 weeks
- More than 12 weeks

[If relevant] Did you have any of the following problems 12 weeks (or more) after catching COVID-19? Please only consider symptoms that are not explained by another reason. Tick all that apply.

- ▪ I was back to my usual self
- ▪ Breathing problems, e.g., breathlessness, pain on breathing, cough
- ▪ Altered sense of taste or smell
- ▪ Problems thinking and communicating e.g., brain-fog, memory problems, difficulty concentrating, decreased alertness, confusion, difficulty speaking
- ▪ Heart problems, e.g., chest pain, palpitation
- ▪ Light-headedness/dizziness on standing
- ▪ Abdominal problems, e.g., tummy pain, diarrhoea, appetite loss
- ▪ Muscle problems, e.g., muscle aches, weakness, severe fatigue
- ▪ Altered feelings in your body, e.g., unusual tingling, pain
- ▪ Problems relating to mood, e.g., anxiety, feeling down, or irritable
- ▪ Problems sleeping, e.g., poor sleep or excessive sleep
- ▪ Skin rashes
- ▪ Bone/joint pain
- ▪ Headaches
- ▪ Other

[If relevant] Thinking of your last, or only, episode of COVID-19, have you now recovered to normal?

- No, I still have some or all of my symptoms
- Yes, I am back to normal
